# Traditional vs Modern: Role of Breed Type in Determining Enteric Methane Emissions from Cattle Grazing as Part of Contrasting Grassland-Based Systems

**DOI:** 10.1371/journal.pone.0107861

**Published:** 2014-09-26

**Authors:** Mariecia D. Fraser, Hannah R. Fleming, Jon M. Moorby

**Affiliations:** Institute of Biological, Environmental and Rural Sciences, Aberystwyth University, Aberystwyth, Ceredigion, United Kingdom; Auburn University, United States of America

## Abstract

Ruminant livestock turn forages and poor-quality feeds into human edible products, but enteric methane (CH_4_) emissions from ruminants are a significant contributor to greenhouse gases (GHGs) and hence to climate change. Despite the predominance of pasture-based beef production systems in many parts of Europe there are little data available regarding enteric CH_4_ emissions from free-ranging grazing cattle. It is possible that differences in physiology or behaviour could influence comparative emissions intensities for traditional and modern breed types depending on the nutritional characteristics of the herbage grazed. This study investigated the role of breed type in influencing CH_4_ emissions from growing beef steers managed on contrasting grasslands typical of intensive (lowland) and extensive (upland) production systems. Using the SF_6_ dilution technique CH_4_ emissions were estimated for a modern, fast-growing crossbred (Limousin cross) and a smaller and hardier native breed (Welsh Black) when grazing lowland perennial ryegrass (high nutritional density, low sward heterogeneity) and semi-improved upland pasture (low/medium nutritional density, high sward heterogeneity). Live-weight gain was substantially lower for steers on the upland system compared to the lowland system (0.31 vs. 1.04 kg d^−1^; s.e.d. = 0.085 kg d^−1^; *P*<0.001), leading to significant differences in estimated dry matter intakes (8.0 vs. 11.1 kg DM d^−1^ for upland and lowland respectively; s.e.d. = 0.68 kg DM d^−1^; *P*<0.001). While emissions per unit feed intake were similar for the lowland and upland systems, CH_4_ emissions per unit of live-weight gain (LWG) were substantially higher when the steers grazed the poorer quality hill pasture (760 vs 214 g kg^−1^ LWG; s.e.d. = 133.5 g kg^−1^ LWG; *P*<0.001). Overall any effects of breed type were relatively small relative to the combined influence of pasture type and location.

## Introduction

The world faces unprecedented challenges with regards to food security for future populations [1]. Ruminant livestock turn forages and poor-quality feeds into human edible products, but there is an inevitable environmental cost in terms of excretion of pollutants [2]–[4]. Methane (CH_4_) is a significant contributor to greenhouse gases (GHGs) and hence to climate change. Agriculture is the source of about 38% of total UK emissions of CH_4_, and of this about 85% comes from livestock enteric sources (mostly ruminants). While beef makes up around 20% of the total meat produced and consumed in the UK, beef cattle account for 27% of the GHG emissions from UK livestock species [3]. Larger, faster-growing animals should theoretically partition relatively more feed nutrients into production and excrete less polluting products during their lifetime, and therefore be more efficient in terms of quantity of product produced. Consequently, the output of polluting excretion products on a per unit product basis should be less for modern cattle breeds than traditional British cattle breeds, which are generally smaller and slower-maturing. However, the latter have frequently been bred under conditions that required them to be hardy and able to survive in exposed conditions on nutritionally poor vegetation [5]. Thus it is possible that physiological or behavioural differences may result in them utilising low-quality native pasture more efficiently than modern breeds. There is also a perception that such breeds have an important role to play in terms of maintaining cultural landscapes [5].

While management-intensive grazing offers potential for more efficient utilisation of grazed forage crops and more efficient conversion of forage into meat and milk [6], within the UK only 12% of meat from cattle is produced in intensive systems [3]. Instead, beef production is predominantly found in areas where physical and climatic challenges limit management options. Over 42% of utilised agricultural land in the UK carries the EU designation of Less Favoured Area (LFA). Among the many factors influencing CH_4_ emissions from ruminants are quantity of feed intake and quality of the diet, with CH_4_ production rising as feed intake increases and as dietary fibre concentrations increase [7]. Consequently emissions would be expected to be lower per unit intake from animals consuming the type of poorer-quality extensively-managed pastures characteristic of farming systems in marginal environments compared to those grazing higher quality cultivated lowland swards, but this has not been directly quantified. Experimental work to date with beef cattle has focussed on the impact of altering feed components within housed systems [8]–[17], and there is a dearth of corresponding data for animals at pasture. The relatively few data which have been collected relate to intensively managed swards [18]–[20] or forage species largely unrepresentative of Western European grasslands [6], [21], [22]. The current study addressed this deficiency, and tested for the first time the role of breed type in influencing CH_4_ emissions from growing beef cattle when pastured on contrasting pasture types representative of intensive (lowland) and extensive (upland) grazing systems.

## Methods

### Ethics statement

The work described was conducted in accordance with the requirements of the UK Animals (Scientific Procedures) Act 1986 and with the approval of the Aberystwyth University Animal Welfare and Ethical Review Board. The conditions under which the animals were studied were designed to be as similar as possible to those used in commercial livestock production systems, and all stock were assessed daily for health and well-being. The research was conducted on one of IBERS’ own research farms (lowland site) and on land leased from the Welsh Government specifically for this research (upland site). All pastures were managed in accordance with EU standards of good agricultural and environmental condition (GAECs).

### Experimental design

Enteric CH_4_ emission values were established for steers of contrasting breed types: a modern, fast-growing cross ((dairy × Belgian Blue) × Limousin) (LimX) and a smaller and hardier traditional breed (purebred Welsh Black) (WB). Separate experimental runs were carried out with animals grazing contrasting pasture types: 1) a lowland monoculture of perennial ryegrass (high nutritional density, low sward heterogeneity), and 2) a semi-improved hill pasture (low/medium nutritional density, high sward heterogeneity).

At the lowland site a total of 4.2 ha of monoculture perennial ryegrass was grazed on a rotational basis. The plots were located 140 m a.s.l. near Aberystwyth, Ceredigion (52°25′43.76″N, 4° 4′9.76″W), and had been sown with perennial ryegrass (cv Premium) in the summer of 2009. The plots were fertilised with 27∶4∶4 (N:P:K) compound fertiliser at a rate of 185 kg ha^−1^ in mid-May 2012, shortly before the start of the experiment.

The upland site grazed consisted of a mosaic of several community types, and was located within the Cambrian Mountains (52°24′5.81′′N, 3°44′0.81′′W), between 525 and 550 m a.s.l. A botanical survey of the 16 ha enclosure was carried out immediately prior to grazing. Around a third of the total area was recorded as being made up of large patches of semi-improved pasture interspersed to varying degrees with *Juncus effusus*. The predominant grass species present within those areas that had been re-seeded decades previously were *Agrostis* spp., *Festuca* spp, *Anthoxanthum odoratum* and *Lolium perenne*. Forbs, mainly *Trifolium repens* and *Cerastium arvense,* were a minor component, accounting for <5% of the sward. At one time these areas had received annual applications of inorganic fertilizer, but no fertiliser had been applied in the two years immediately preceding the experiment. The remaining two-thirds of vegetation within the enclosure consisted of patches of Blanket Bog Priority Habitat, Purple Moorgrass and Rush Pastures Priority Habitat [23], and dense *J. squarrosus*. When grazing the hill pasture the animals had access to the entire enclosure for the duration of the experiment.

Groups of steers born March – May 2011 were selected for each experimental run based on uniformity of age, body condition score (BCS) [24] and within-breed live weight (n = 9 steers per breed/system combination). All animals were drenched with an anthelmintic prior to the start of grazing. The lowland ryegrass experimental run commenced on 28 June 2012, and the hill sward experimental run on 23 July 2012. The later start at the upland site reflected the three-week or so delay in the start of the growing season and subsequent timing of peak growth at this location relative to the coastal lowland site. Each experimental run consisted of three phases: an adaptation phase, a performance measurement phase, and a CH_4_ measurement phase. Following turnout onto the experimental pastures the animals were given at least two weeks to adapt to the site and pasture before data collection began. There then followed a six-week performance measurement period during which live weight was recorded weekly in order to establish individual growth rates. During a subsequent two-week CH_4_ measurement period associated enteric emissions of CH_4_ were then estimated. The animals at each site grazed together as a single group to ensure that opportunities for selective grazing were similar for the two breeds when grazing the heterogeneous hill sward.

### Sward measurements

Sward height and biomass data were collected weekly to monitor herbage availability. Sward height was measured at each site using a sward stick (50 measurements per plot) [25]. At each measurement location the height of the first touch of grass/forb vegetative growth was recorded. Herbage biomass samples were collected by cutting the material along a 1 m rule to ground level at 10 random locations across each site using electric shears (ryegrass; 2 cuts per location) or a hedge-trimmer (hill sward; 1 cut per location). In order to reflect availability of preferred vegetation, sampling on the hill sward was restricted to areas when the cattle had been observed as grazing; generally the patches of semi-improved pasture. Following weighing of the fresh cut material a representative sub-sample was oven dried (100°C) to constant weight to determine dry matter (DM) content. The remaining herbage from each sampling location was bulked into a single weekly sample per site. A sub-sample of this bulked material was subsequently freeze-dried and milled to pass through a 1 mm sieve prior to chemical analysis. Ash was measured by igniting samples in a muffle furnace at 550°C for 16 h, and gross energy (GE) was determined by adiabatic bomb calorimetry (Gallenkamp autobomb; Sanyo Gallenkamp PLC, Loughborough UK). Total nitrogen (TN) concentrations were determined using a Leco FP 428 nitrogen analyser (Leco Corporation, St. Joseph, MI, USA), and expressed as crude protein (CP) (TN × 6.25). Water-soluble carbohydrate (WSC) concentrations were measured by an automated anthrone technique [26]. Neutral-detergent fibre (NDF) and acid-detergent fibre (ADF) were determined using the method of Van Soest *et al*. [27], adapted for the Gerhardt Fibrecap detergent system (FOSS UK Ltd, Warrington, UK). Digestibility of organic matter in the DM (DOMD) was determined using the two-stage pepsin-cellulase *in-vitro* method described by Jones & Hayward [28].

### Animal measurements

The live weights and BCS of the animals were recorded once weekly throughout each grazing session. Incremental live-weight gains were calculated and averaged across the performance measurements period on an individual animal basis. Assessments of BCS were made using a scale from 1 to 5 [24], with quarter scores as intermediate points along the scale.

Enteric CH_4_ emissions were estimated using the sulphur hexafluoride (SF_6_) tracer technique as described by Muñoz *et al*. [29]. A brass permeation tube with known SF_6_ release rate was inserted *per os* into the reticulo-rumen of each steer prior to turn-out onto the experimental pastures. The release rates of the permeation tubes used averaged 4.773 mg d^−1^. Breath was sampled from each steer via an inlet mounted on a halter and located above the nose. All animals were allowed at least a week to adapt to wearing the equipment prior to sample collection. Within the collection period samples were collected for 4 d for each animal during two consecutive weeks. Animals were fitted with a 1.7 l collection canister, previously evacuated to>90 kPa pressure and fitted with a capillary tube previously prepared to provide gaseous collection at a rate of between 0.35 and 0.45 ml min^−1^. The time between changing collection canisters was as close to 24 h as possible. In order to record ambient CH_4_ and SF_6_ concentrations two additional canisters were placed close to each grazing area, but away from the animals. These were replaced every 24 h as for each animal collection canister. After each 24 h collection period, the residual vacuum pressure was recorded for each canister. The canisters were then pressurised with nitrogen to approximately 50 kPa prior to analysis by gas chromatography within 48 hrs. Methane and SF_6_ concentrations were quantified using a gas chromatograph (Clarus 560; PerkinElmer, Cambridge, UK) fitted with a flame ionisation detector and an electron capture detector. The sample inlet was connected to a 1 ml sample loop via a valve, which at the initiation of each analytical run delivered the sample via a T connection to two packed stainless steel columns: 1.219 m × 3.175 mm OD × 2 mm ID 80/100 Porapak N for CH_4_ analysis, and 1.829 m × 3.175 mm OD × 2 mm ID 45/60 molecular sieve 5A, for SF_6_ analysis (both columns sourced from Sigma-Aldrich Company Ltd, Gillingham, Dorset, UK). The carrier gas was oxygen-free nitrogen with a flow rate of 40 ml min^−1^, split between the two columns; air and hydrogen were supplied to the flame ionisation detector at the rates of 450 ml min^−1^ and 45 ml min^−1^ respectively. The oven temperature was a constant 70°C, and the heater temperature on both detectors was set at 250°C. Total run time was 1.30 min. Calibration curves for quantification were prepared using standard gas mixtures in nitrogen (Scott-Marin, Inc, Riverside CA, USA): 1) 10.25 ppmv CH_4_ (±1% NIST) and 9.43 pptv SF_6_ (±10% NIST); 2) 102.9 ppmv CH_4_ (±1% NIST) and 146 pptv SF_6_ (±5% NIST); and 3) 307 ppmv (±1% NIST) and 295 pptv SF_6_ (±5% NIST). A fourth standard mixture (51.3 ppmv CH_4_, ±1% NIST, and 81.8 pptv SF_6_, ±5% NIST) was used as a quality assurance standard during sample analysis runs.

Methane emissions (g d^−1^) from each individual animal were calculated from the measured SF_6_ and CH_4_ concentrations sampled by the canisters (SF_6_C and CH_4_C respectively), background concentrations of SF_6_ and CH_4_ (SF_6_B and CH_4_B respectively) and the release rate of SF_6_ (SF_6_R, in g d^−1^) from individual permeation tubes determined before the start of the experiment according to Equation 1.
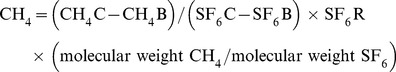
(1)


### Climatic conditions

During the CH_4_ sampling periods at each site wind speed was measured using a yacht anemometer (Type 454; Schiltknecht Messtechnik AG, Gossau, Switzerland) fixed approximately 1.5 m from ground level and connected to a battery powered datalogger (MSR 145; MSR Electronics GmbH, Seuzach, Switzerland) that also recorded atmospheric pressure, temperature and relative humidity at 30 second intervals. The datalogger was housed in a standard Stevenson screen located at the edge of the experimental plots. Data relating to measured rainfall were obtained from the meteorological station nearest to each experimental site.

### Data analysis

The effect of breed type and production systems on animal performance was investigated with individual animal as the experimental unit. Feed intake was estimated by calculating metabolisable energy (ME) requirements for measured live-weight gain using AFRC [30] equations. The GE density of CH_4_ used was 55.65 MJ kg^−1^, and feed GE density was as analysed in samples collected (17.53 and 18.65 MJ kg^−1^ DM for lowland and upland pastures respectively). The metabolisability of feed GE at maintenance (qm) was calculated from sward sample ME values, with forage ME being calculated as 2.34+0.0111 × DOMD [30]. Energy requirements for maintenance and growth were estimated from mean live weight and live-weight change respectively, with mean scaling factors (C2) of 1.15 and 1.0 for LimX and WB cattle respectively, to account for differences in the maturing age of the breeds [30]. The DM intake required to supply ME requirements was calculated using predicted feed ME density.

Data relating to live weight, BCS, growth and CH_4_ emissions were analysed using analysis of variance with a treatment structure of breed type (WB, LimX) × system (Lowland, Upland). In this context ‘system’ was used as a collective term for the combination of factors relating to sward, climate and terrain which potentially influenced the nutritional demands and grazing behaviour of the animals at each site. One LimX steer on the lowland system had to be excluded from the study on behavioural grounds, and two LimX steers on the upland systems had to be excluded on health grounds. These animals were treated as missing values in the analysis. Tier 1 [31] equivalent emission factors (EFs) (kg y^−1^) were calculated as: CH_4_ (g d^−1^) x 365.

## Results

### Climatic conditions

Mean temperatures recorded during the CH_4_ collection period at the lowland site were considerably higher those recorded at the upland site, while average wind speeds recorded were broadly similar, with an identical range (Table 1). Mean relative humidity was lower at the lowland site than at the upland site, with similar ranges. Based on data from the nearest meteorological stations to the two sites, rainfall was estimated to be substantially higher at the upland site across the experimental periods (193 vs 104 mm respectively). Differences in atmospheric pressure between the two sites reflected the differences in altitude.

**Table 1:** Meteorological data recorded during the methane data collection periods. Values are means (minimum – maximum).

	Lowland	Upland
Temperature (°C)	14.2 (5.8–18.0)	6.9 (2.3–11.1)
Wind speed (km h^−1^)	8 (0–44)	11 (0–44)
Atmospheric pressure (kPa)	99.8 (98.1–101.6)	94.4 (92.1–96.0)
Relative humidity (%)	87 (60–99)	94 (66–100)

### Sward characteristics

The mean sward surface height of the grazed ryegrass sward across the 8 weeks of data recording was 12 cm (s.e. = 0.9 cm), with the corresponding mean herbage biomass 1570 kg ha^−1^ (s.e. = 117 kg ha^−1^). The mean sward surface height of the grassy (i.e. non-*Juncus*) areas of the hill sward was 16 cm (s.e. = 2.2 cm), and the mean herbage biomass for these areas was 2740 kg ha^−1^ (s.e. = 281 kg ha^−1^). These results indicate that performance would be limited by the quality rather than the quantity of herbage available, particularly at the upland site.

The herbage cut from the upland site was characterised by having lower CP and WSC concentrations relative to the material collected from the lowland site, and a lower DOMD (Table 2). The high ash concentration for the ryegrass cut from the lowland site likely reflects the higher than average rainfall during the summer of 2012 [32] and associated soil contamination during cutting.

**Table 2:** Chemical composition of swards available to steers on contrasting beef production systems (where Lowland = rotational grazing of monoculture perennial ryegrass, Upland = extensive grazing of a diverse hill sward).

	Lowland	Upland
DM (g kg^−1^)	221 (17.0)	203 (16.0)
Ash	119 (16.1)	33 (1.6)
CP	148 (6.7)	120 (5.2)
WSC	126 (9.8)	94 (6.4)
NDF	508 (17.8)	677 (5.8)
ADF	283 (11.1)	363 (8.6)
DOMD	591 (8.9)	502 (5.0)
GE (MJ kg^−1^ DM)	17.5 (0.40)	18.7 (0.35)

Values are means (with standard errors) of eight weekly samplings across the measurement period at each site. All values g kg^−1^ DM unless otherwise stated.

### Animal performance

In keeping with what would be expected for a native breed type, the WB steers were smaller than the LimX steers at the start of data recording at both sites (Table 3). The BCS of the two breeds was similar however (grand mean = 2.5).

**Table 3:** Effects of breed type and production system on animal performance and methane (CH_4_) emissions of beef steers (where WB = Welsh Black, LimX = Limousin cross; Lowland = rotational grazing of monoculture perennial ryegrass, Upland = extensive grazing of a diverse hill sward).

	Lowland		Upland			*F* prob.^2^	
	WB	LIM	WB	LIM	s.e.d.	Breed	System
Start weight (kg)	386	458	410	467	12.7	<0.001	ns
BCS at start	2.5	2.4	2.4	2.4	0.08	ns	ns
Estimated DM intake (kg d^−1^)	10.3	11.8	7.7	8.2	0.67	0.05	<0.001
CH_4_ (g d^−1^)	216	217	173	190	10.4	ns	<0.001
EF^1^ (kg year^−1^)	79	79	63	69	3.8	ns	<0.001
CH_4_/DM intake (g kg^−1^)	21.0	18.7	22.9	23.4	1.33	ns	<0.01
CH_4_-E/GE intake (%)	6.7	6.0	6.8	7.0	0.41	ns	<0.05

1 Calculated for comparison with IPCC [31] Tier 1 default values; ^2^F probability; ns = non-significant, *P*>0.1; there were no significant interaction effects.

Where BSC = body condition score; EF = Emission Factor; GE = gross energy.

**Figure 1:**
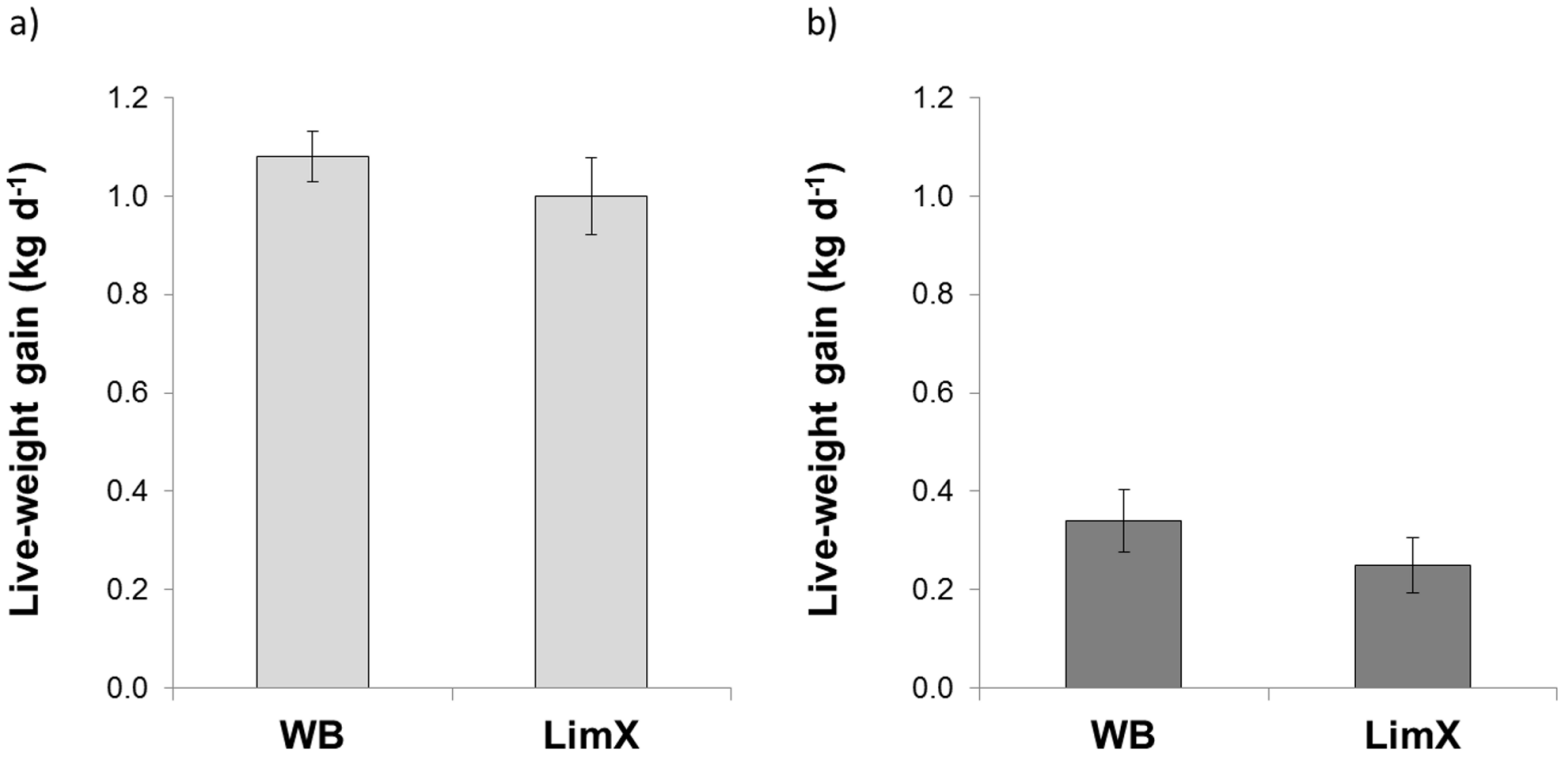
Effect of breed type on growth rates of steers grazing on a) lowland ryegrass, and b) semi-improved hill pasture. (Where WB = Welsh Black and LimX = Limousin cross; values plotted are mean±standard errors).

Pasture type had a highly significant effect on live-weight gain (s.e.d. = 0.08 kg d^−1^; *P*<0.001), which was just over 1 kg d^−1^ when grazing the lowland ryegrass sward, but less than 0.35 kg d^−1^ when grazing the hill sward (Fig. 1). Overall the growth rates for the two breed types were similar, and there were no breed type × pasture type interaction effects. There was a trend towards estimated DM intakes being influenced by breed type, but once again the results obtained were more strongly influenced by system type, with the steers grazed on the upland site estimated to consume substantially less forage than those grazed on the lowland site (Table 2).

### Methane production

**Figure 2:**
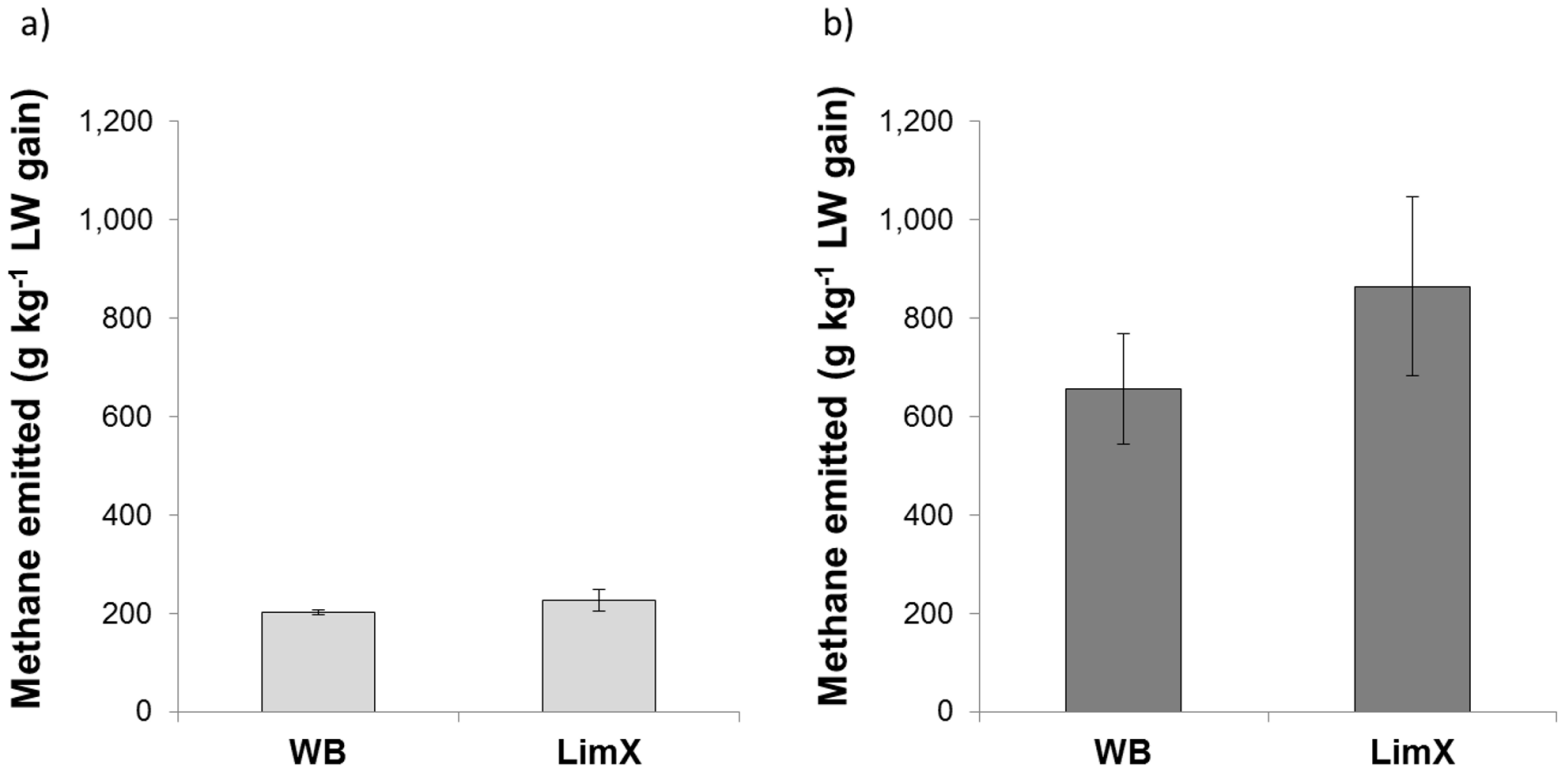
Effect of breed type on methane emissions per kilogram of live-weight (LW) gain for steers grazing on a) lowland ryegrass, and b) semi-improved hill pasture. (Where WB = Welsh Black and LimX = Limousin cross; values plotted are mean±standard errors).

When the effects of breed type and system were analysed, CH_4_ yield was significantly lower for animals on the upland system (Table 3). Neither breed type nor system influenced the amount of CH_4_ emitted per unit of feed consumed. Likewise, the yield of CH_4_ energy per unit GE intake (Y_m_) was similar for both systems, with the grand mean 6.0%. Emissions intensities (CH_4_ emitted per unit weight gain) were significantly lower for steers on the lowland system (s.e.d. = 133 g kg^−1^ live-weight gain; *P*<0.001) compared to the upland system (Fig. 2). There was also considerably more between-animal variation recorded for steers of both breed types on the upland compared to the lowland system.

## Discussion

Improvements in production efficiency have the potential to decrease the carbon footprint of livestock product [3]. Increasing the proportion of concentrates in the diet generally reduces CH_4_ emissions, both as a proportion of energy intake and when expressed per unit of meat or milk output [33]. Dawson [34] compared the carbon footprint of a long-keep steer system (in which cattle were offered grazed grass in the summer and grass silage in the winter) with that of an intensive bull system (in which bulls were housed throughout their lives and offered concentrates *ad libitum*), and showed that the carbon footprint of the bulls expressed as CO_2_e per kg carcass weight was approximately half that of the steers. The challenge to grass-based livestock systems is therefore to strive for levels of animal performance that are comparable to those associated with intensive cereal-based systems.

### Comparative performance of different breed types under lowland and upland conditions

There was no effect of breed type on the performance figures recorded for either production system. In previous years the hill enclosure had been mixed grazed by sheep and suckler cows during the summer months as part of commercial farm operations, and as such the swards were expected to support productive stock. Despite the expectation that selective grazing of the improved areas within the enclosure would raise the nutritional value of the diets consumed relative to the average for the sward as a whole, the growth rates achieved by both breeds during the current study were low and not dissimilar to those previously recorded for yearling cattle when grazing another site in the area that was entirely dominated by the native hill grass species *Molinia caerulea* [35]. Likewise the growth rates are considerably lower than the figure of 0.64 kg d^−1^ recently reported for dairy-cross steers grazing botanically diverse upland pasture at a lower altitude in Northern Ireland [36]. It would therefore seem that withdrawal of fertiliser inputs had already had a deleterious effect on the species composition and therefore the nutritional quality of the sward despite ryegrass and white clover still being in evidence [37]. In the longer term, further reductions in the competitive ability of the sown species would be expected. At the same time, ceasing applications of inorganic fertiliser would be expected to reduce net nitrous oxide emissions from the production system. Further research is required to build on these baseline data and simultaneously test the impact of such management practices on carbon and nitrogen capture and loss in order to assess overall system efficiency.

During the current experiment voluntary feed intake was estimated by back calculation based on the predicted energy requirements of the stock to achieve the performance recorded and the corresponding nutritional value of the sward being consumed. However, a range of factors may have influenced the demands of the animals. In particular the exposed nature of the upland site would have placed additional burdens on the animals. Climatic conditions (temperature, rainfall, windspeed etc) are not taken into account by the AFRC [30] energy requirement calculations. The thermoneutral zone of cattle is generally considered to be greater than that of other livestock [38], although it is affected by coat depth, coat conditions (wet, muddy etc) and wind speed [39]. Even though the lower critical temperature for beef cattle is estimated to be about −21°C in still dry conditions [38], energy requirements increase to support metabolic heat production in wet beef cattle at temperatures as high as 15°C [40]. At the same time, high wind speeds coupled with the animals being frequently wet from rainfall, particularly at the upland site, may have altered grazing behavior and led to reductions in grazing time as the animals sought shelter.

When estimating intake qm was calculated using predicted ME values of sward samples. While the sward samples were taken from patches preferentially grazed by the cattle, they will not have reflected within-patch selection of particular sward components which may have led to the diet consumed having a higher digestibility than the average of the sward on offer. Furthermore, *in vitro* digestibility estimations based on enzyme preparation do not leave any scope for possible interaction between microbial species in the rumen and the modification of this by the diet of the host animal [41]. This will have likely led to the calculated figures overestimating actual feed intake. This would mean that the estimates of CH_4_ yield from feeds and Y_m_ are lower than the true values, and may be altered to some extent if actual DM and GE intakes were known. Despite this, the values of Y_m_ measured in this study are similar to those reported by other studies for grazing cattle [42]–[44]. Alternative marker-based methods of estimating feed intake such as the *n*-alkane technique [45] also have limitations, particularly when used in situations where it is difficult to obtain a representative sample of the diets selected from a heterogeneous sward. Another approach to measuring intake and CH_4_ emissions is housing the animals in respiration chambers and offering them cut forage. However, crucially this significantly reduces any environmental effects on productive and excretion outputs, and prevents the animals from exhibiting normal grazing behaviour. Therefore, it was felt that the methods employed in this study were most appropriate to obtain the data collected, and any deviation of estimates from absolute values are likely to be small while relative differences are comparatively precise.

### Comparative enteric emissions under lowland and upland conditions

There are previous reports of CH_4_ emissions differing on different forage types [18], and CH_4_ emissions per unit carcass gain have been shown to decrease as pasture quality improves [46]. Consequently it is not surprising that the emissions per kg live-weight gain from the cattle grazing the poorer quality pasture within the upland system are higher. While differences between pastures types are confounded with environmental conditions within the present study, the values recorded are representative of the grazing system as a whole. Zero-grazing of the different swards would have allowed the influence of climatic conditions to be controlled, but crucially the role of foraging strategy in influencing emissions would also have been negated. Selective feeding can lead to the nutritive value of a diet consumed by animals grazing heterogeneous swards being substantially higher than the average for the sward as a whole. Furthermore, it was possible that breed differences in the composition of the diet selected by the steers grazing the hill pasture could be reflected in differing CH_4_ emissions. The overall similarity in emissions for the two breeds would however suggest that there were no substantial differences in the diets chosen, in keeping with the findings of previous research on breed differences in cattle grazing preferences [5], [47]. Although the WB steers were smaller than the LimX steers they are a comparatively large native breed. Previous calculations estimating potential CH_4_ emissions from suckler cows with calves at foot suggested that CH_4_ emissions per kilogram of calf weight gain would be higher for smaller Belted Galloway cattle compared to Limousin-crosses [48]. This difference in predicted relative performance of a traditional breed was due to lower absolute weight gains by the Belted Galloway calves, despite them having higher proportional gains per kilogram initial weight.

The current UK National GHG Inventory largely reports emissions from agriculture to the United Nations Framework on Climate Change using the most simplified approach to accounting (i.e. Tier 1 methodology). This methodology uses generic assumptions and factors about livestock management to estimate GHG emissions, and relies on default EFs published by the IPCC [31]. The EF quoted by the IPCC for non-dairy cattle in Western Europe is 48 kg head^−1^ yr^−1^, and is applicable to bulls, calves and growing steers or heifers. The equivalent EFs calculated from the current experimental work are higher than this, although it must be noted that the values reported here are representative of values achieved under summer grazing only rather than the production system as a whole. The inclusion of grain-based diets within the winter feeding phase could reduce the overall emission burden [34]. The values obtained provide a valuable contribution towards the development of the necessary evidence base for the UK and other countries with similar temperate grassland systems of beef production to move to the more complex Tier 2 and Tier 3 approaches for reporting livestock emissions.

### Wider implications

Many upland areas can, with appropriate nutrient inputs, sustain moderate levels of animal performance and although the carbon footprint per kilogram of carcass will be higher relative to lowland intensive systems, there are benefits both for human health and for food security from grass-based meat production [49], particularly when forage from areas unsuitable for cultivation are turned into human-edible products. Furthermore, the vegetation communities found in the hill and uplands support a variety of ecosystem services, such as biodiversity and landscape character, which are frequently dependent upon livestock farming. The UK, European and worldwide importance of the associated habitats in terms of nature conservation is recognised under legislation such as the EU Habitat and Bird Directives. The management of priority habitats such as those included within the hill enclosure used during the current experiment are frequent targeted by options within higher level agri-environment schemes. However, the results from the current study confirm that the associated conservation strategies designed to enhance biodiversity result in increased GHG emissions per unit of product due to the poorer quality of the vegetation consumed. Thus they make an important addition to the evidence base for future policies relating to Areas of Natural Constraint (ANC); the new designation due to replace LFA shortly. Further multi-disciplinary research is required to quantify and explore the trade-off between biodiversity and other ecosystem benefits and GHG emissions arising from grazing of semi-natural vegetation communities. This is of particular relevance to situations where incentives are being used to encourage the re-introduction of grazing to abandoned or under-utilised pasture. While CH_4_ emission intensities would be expected to be relatively high in the first instance, these may lower as grazing rejuvenates the pasture and related changes in plant morphology or species balance within the sward lead to an improvement in nutritional value.

Although dietary strategies such as supplementation with fat, higher starch diets, use of monensin, exogenous enzymes and direct-fed microbials are being evaluated as potential means of reducing enteric CH_4_ emissions [50], options for deploying such strategies are limited in extensive grazing systems. Further research is also required to develop and test alternative or modified strategies for manipulating rumen microbial populations as a means of reducing CH_4_ from free-ranging grazing animals.

## Conclusions

The current study was the first to quantify enteric CH_4_ emissions for free-ranging beef cattle pastured on these common grassland types. It has shown that CH_4_ emission intensities for growing steers at pasture are more strongly influence by production system than breed type, and established that emissions per unit of live-weight gain are substantially higher for animals grazed extensively on semi-improved hill pasture than animals grazing lowland ryegrass swards. Breed had comparatively little impact on the results obtained, and any numerical differences observed are likely to be caused by differences in feed intake. The data generated will strengthen the comparatively limited evidence base for future policy development regarding climate change mitigation and adaptation strategies within pastoral livestock systems.
